# The Influence of Rare Earth Ce on the Microstructure and Properties of Cast Pure Copper

**DOI:** 10.3390/ma17102387

**Published:** 2024-05-16

**Authors:** Mingyi Zhang, Jichun Yang, Haixiao Li

**Affiliations:** 1 School of Rare Earth Industry, Inner Mongolia University of Science and Technology, Baotou 014010, China; 15384850013@163.com; 2 Key Laboratory of Green Extraction & Efficient Utilization of Light Rare-Earth Resources (Inner Mongolia University of Science and Technology), Ministry of Education, Baotou 014010, China; 3 Inner Mongolia Institute of Metal Materials, Baotou 014034, China; 4 Xinzhou Comprehensive Inspection and Testing Center, Xinzhou 034000, China; 18734170553@163.com

**Keywords:** pure copper, rare earth, organization, performance, thermodynamics

## Abstract

The effects of rare earth Ce on the microstructure and properties of cast pure copper were investigated through thermodynamic calculations, XRD analysis, mechanical testing, metallographic microscopy, and scanning electron microscopy (SEM). The experimental results demonstrate that the reaction between rare earth Ce and oxygen as well as sulfur in copper exhibits a significantly negative Gibbs free energy value, indicating a strong thermodynamic driving force for deoxidation and desulfurization reactions. Ce is capable of removing trace amounts of O and S from copper. Moreover, the maximum solid solubility of Ce in Cu falls within the range of 0.009% to 0.01%. Furthermore, Ce can refine columnar grains while enlarging equiaxed grains in as-cast copper. Upon the addition of rare earth Ce, the tensile strength increased by 8.45%, elongation increased by 12.1%, and microhardness rose from 73.5 HV to 81.2 HV—an increase of 10.5%. Overall, rare earth Ce has been found to enhance both the microstructure and mechanical properties of cast pure copper.

## 1. Introduction

Pure copper exhibits excellent electrical and thermal conductivity, high ductility, and exceptional processability, making it extensively utilized in various fields such as the electronic industry, the electric power sector, and military applications [1,2,3,4]. The attainment of copper material with elevated cleanliness levels, a uniform microstructure, and superior properties is crucial to ensure the subsequent processing procedures and product quality.

The application of rare earth in pure copper has been relatively under-reported compared to its extensive research and excellent effects observed in iron and steel materials, aluminum, magnesium, and other alloy materials [5,6,7,8]. Existing studies suggest that rare earth can also enhance the microstructure and properties of copper alloys. Rare earth primarily functions in copper alloys by the following: (i) exhibiting highly reactive chemical properties that enable it to react with detrimental elements such as oxygen, sulfur, hydrogen, etc., forming high-melting-point compounds which float on the slag and act as purifying agents [9,10,11] and (ii) dispersing fine high-melting-point compounds throughout the copper alloy matrix to serve as new crystalline cores for grain refinement [12,13,14]. Due to its reactivity, rare earth readily reacts with impurities in copper to form well-defined shape-rich compounds with high melting points, thereby improving the morphology of original inclusions [15,16]. Consequently, rare earth significantly alters the existing forms of impurities within copper alloys while remarkably enhancing their properties.

Considering the advantageous impact of rare earth elements on copper alloys, this study aims to investigate the influence of Ce on the microstructure and mechanical properties of pure copper. The objective is to ascertain whether rare earth elements have a similar effect on pure copper, thereby enhancing the overall characteristics of cast pure copper.

## 2. Materials and Methods

The cathode copper, serving as the experimental raw material, is melted using a 25 Kg SK-NL300 vacuum medium-frequency induction furnace at a temperature of 1200 °C. In order to take advantage of the low oxidation tendency and similar melting point to Cu exhibited by the Ce-Cu alloy, rare earths are incorporated in the form of a Ce-Cu intermediate alloy. The content of rare earth element Ce in the Ce-Cu alloy amounts to 35%. By employing an Inductively Coupled Plasma Mass Spectrometer (ICP-MS), the rare earth content in the melted test copper is measured, resulting in values of 0 ppm and 97 ppm for sample numbers 1# and 2#, respectively. Thermodynamic calculations were initially employed to analyze the reaction between rare earth element Ce and typical harmful elements present in copper from a theoretical perspective, thereby determining the reaction products.

The organization and performance of the test samples were evaluated using the following methods: The columnar and equiaxed grain regions of the copper ingots after smelting were observed under a low-magnification microscope, specifically the Axiocam105 color model. The crystal structure and phase analysis of the samples were conducted using a Rigaku SmartLab SE X-ray diffractometer (Manufactured by Rigaku Corporation, Tokyo, Japan) with a scanning range from 10° to 80° at a scanning speed of 2°/min. A microstructure observation and EDS spectrum analysis of the samples were performed using a TESCAN MIRA LMS electron microscope (SEM) (The equipment was manufactured by TESCAN, Brno, Czech Republic) operating at an acceleration voltage ranging from 200 V to 30 KV, a probe current ranging from 1 pA to 100 nA, with stability better than 0.2%/h. Hardness testing was carried out on the samples using a KSV-2500 microhardness tester with a loading load of 0.2 Kg (200 gf). According to the standard (GB/T228.1-2021 [17]), the tensile performance of the specimen was evaluated using a CMT5305 electronic universal testing machine (Manufactured by MTS Systems Corporation in Shanghai, China).

## 3. Results

### 3.1. Thermodynamic Investigation of Primary Ce-Containing Inclusions in Cast Pure Copper

Although the impurity elements in pure copper are present at very low levels (less than 0.01%), these trace impurities can significantly impact the properties of pure copper. For instance, the formation of brittle compounds such as Cu_2_O and CuS due to the presence of oxygen and sulfur can greatly reduce its plasticity. Additionally, rare earth elements exhibit a strong affinity for oxygen and sulfur, leading to the formation of high-melting-point rare earth compounds with excellent thermal stability and low specific gravity, thereby playing a crucial role in purifying liquid copper. Understanding the thermodynamics of rare earth reactions within copper serves as a fundamental basis for investigating their influence on this metal. The occurrence of Ce-containing inclusions in cast pure copper can be explained from a thermodynamic perspective.

#### 3.1.1. Thermodynamic Conditions and the Sequential Formation of Ce-Containing Impurities

At a temperature of 1200 °C, the presence of the rare earth element Ce in copper can lead to the formation of various reaction products. By utilizing the corresponding thermodynamic data [18,19,20,21], it becomes possible to predict the thermodynamic conditions and sequence of formation for rare earth impurities in copper through reactions between Ce and main elements O and S. Table 1 presents the reaction products as well as the activity product of Ce with O and S in liquid copper.

At a temperature of 1200 °C, the activity coefficient of rare earth Ce, when added to the main elements in the multi-component copper system, is as follows [20]: ƒ_Ce_ = 0.9890, ƒ_o_ = 0.4537, and ƒ_s_ = 0.6405. In this experiment, $\omega_{\left[Ce\right]}=0.0097\%$,$\;\omega_{\left[O\right]}=0.0003\%$, and $\omega_{\left[S\right]}=0.0005\%$. Therefore, the activity of Ce, S, and O in the entire group of copper elements can be obtained: α_Ce_ = 9.6 × 10^−3^, α_O_ = 1.4 × 10^−4^, and α_S_ = 3.2 × 10^−4^_._

(1) The thermodynamic conditions governing the interconversion between CeO_2_ (s) and Ce_2_O_3_ (s)

According to the reaction 

Ce_2_O_3_(s) + [O] = CeO_2_ (s) (1)

(2) $$∆G^{\theta }=RTln{\left(\frac{\prod a_{1}}{\prod a_{2}}\right)}^{2}$$

Derived from the chemical isothermal equation as follows:

(3) $$∆G=∆G^{\theta }+RTln\frac{1}{a_{0}}=RTln\frac{{\left(\prod a_{1}\right)}^{2}}{{\left(\prod a_{1}\right)}^{2}\cdot a_{0}}$$

When ∆G < 0, indicating $\frac{{\left(\prod a_{1}\right)}^{2}}{{\left(\prod a_{1}\right)}^{2}\cdot a_{0}}\;$< 1 or $a_{0}$ > 1.89 × 10^−4^, the reaction proceeds towards the formation of CeO_2_ (s); otherwise, it leads to the production of Ce_2_O_3_ (s). In this experimental study, as $a_{0}$ = 1.4 × 10^−4^ < 1.89 × 10^−4^, the resulting product is determined to be Ce_2_O_3_.

(2) The thermodynamic conditions governing the interconversion between Ce_2_O_3_ (s) and Ce_2_O_2_S (s)

According to the reaction

Ce_2_O_3_ (s) + [S] = Ce_2_O_2_S (s) + [O] (4)

Derived from the chemical isothermal equation as follows:

(5) $$∆G=∆G^{\theta }+RTln\frac{a_{0}}{a_{s}}=RTln\frac{{\left(\prod a_{3}\right)}^{2}\cdot a_{0}}{{\left(\prod a_{2}\right)}^{2}\cdot a_{s}}$$

When ∆G< 0, i.e., when $\frac{{\left(\prod a_{3}\right)}^{2}\cdot a_{0}}{{\left(\prod a_{2}\right)}^{2}\cdot a_{s}}$ < 1 or $\frac{a_{0}}{a_{s}}<\frac{{\left(\prod a_{2}\right)}^{2}}{{\left(\prod a_{3}\right)}^{2}}\;$= 0.4374, the reaction proceeds towards the right to form Ce_2_O_3_ (s); conversely, it yields Ce_2_O_2_S (s). In this experiment, $\frac{a_{0}}{a_{s}}\;$= 0.4375 and $\frac{{\left(\prod a_{3}\right)}^{2}\cdot a_{0}}{{\left(\prod a_{2}\right)}^{2}\cdot a_{s}}$ = 1.0002 ≈ 1, thus indicating an equilibrium reaction where both Ce_2_O_3_ (s) and Ce_2_O_2_S (s) are produced.

(3) The thermodynamic conditions governing the interconversion between CeS (s) and Ce_2_S_3_ (s)

According to the reaction

2CeS (s) + 2[S] = Ce_2_S_3_ (s) (6)

Derived from the chemical isothermal equation as follows:

(7) $$∆G=∆G^{\theta }+RTln{\left(\frac{1}{a_{s}}\right)}^{2}=RTln\frac{{\left(\prod a_{5}\right)}^{2}}{{\left(\prod a_{4}\cdot a_{s}\right)}^{2}}$$

When ∆G < 0, indicating $\frac{{\left(\prod a_{5}\right)}^{2}}{{\left(\prod a_{4}\cdot a_{s}\right)}^{2}}$ < 1 or $a_{s}>\frac{\prod a_{5}}{\prod a_{4}}\;$= 0.7737, the reaction proceeds towards the formation of Ce_2_S_3_(s); otherwise, it leads to the production of CeS (s). In this experimental study, as $a_{s}$ = 3.2 × 10^−4^ < $\frac{\prod a_{5}}{\prod a_{4}}\;$= 0.7737, the resulting product is determined to be CeS (s).

(4) The thermodynamic conditions governing the interconversion between CeS (s) and CeS_4_ (s)

According to the reaction

CeS (s) + 3[S] = CeS_4_ (s) (8)

Derived from the chemical isothermal equation as follows:

(9) $$∆G=∆G^{\theta }+RTln{\left(\frac{1}{a_{s}}\right)}^{3}=RTln\frac{\prod a_{6}}{\prod a_{4}\cdot {\left(a_{s}\right)}^{3}}$$

When ∆G < 0, indicating $\frac{\prod a_{6}}{\prod a_{4}\cdot {\left(a_{s}\right)}^{3}}$ < 1 or ${\left(a_{s}\right)}^{3}>\frac{\prod a_{6}}{\prod a_{4}}$ = 0.2776 × 10^2^, the reaction proceeds towards the formation of CeS_4_ (s); otherwise, it leads to the production of CeS (s). In this experimental study, as ${\left(a_{s}\right)}^{3}<\frac{\prod a_{6}}{\prod a_{4}}$, the resulting product is determined to be CeS (s).

According to the thermodynamic conditions calculated based on the aforementioned interconversion of reaction products, it can be inferred that in this experimental study, the final inclusion compounds formed by rare earth Ce in a copper melt at 1200 °C consist of Ce_2_O_3_ (s), Ce_2_O_2_S (s), and CeS (s).

#### 3.1.2. Thermodynamic Properties of Cu-Ce-O System

The reaction between rare earth cerium and oxygen in liquid copper at 1200 °C can be described as follows:

(10) $$[\mathrm{Ce}{]}_{\mathrm{Cu}}+\frac{3}{2}[O]{\mathrm{Cu}}_{\;}=\frac{1}{2}{\mathrm{Ce}}_{2}O_{3}(s)\;\;G_{1}^{\theta }\;=\;-1,074,469\;+\;415.28T\;=\;-462,761.56$$

(11) $$∆G=∆G^{\theta }\;+\;\mathrm{RTlnJ}$$

∆G is the change in the Gibbs free energy of the chemical reaction; ∆G^θ^ is the standard Gibbs free energy; J is activity quotient; R is the gas constant; T is the Kelvin temperature.

Taking a 1% solution by mass as the standard state, the calculation formula of activity α_i_ is as follows:

α_i_ = f_i_ω_i_ (12)

(13) $$lgf_{i}=\sum_{j=1}^{n}e_{i}^{j}\omega_{\left[j\right]}$$

Ce_2_O_3_ is a pure material, so $\alpha_{{Ce}_{2}O_{3}}$ = 1 during the calculations is the activity interaction coefficient of component j to component i in copper liquid. The interaction coefficient between main elements in copper is shown in Table 2. At 1200 °C, because the activity of O is extremely low, it can be considered that $\alpha_{{Ce}_{2}O_{3}}$. In the formula $\omega_{\left[Ce\right]}=0.0097\%$, $\omega_{\left[O\right]}=0.0003\%$, and $\omega_{\left[S\right]}=0.0005\%$, the values were measured in the test, and the average value of multiple measurements was taken.

(14) $$∆G_{1}\;=∆G_{1}^{\theta }\;+\mathrm{RTlnJ}=∆G_{1}^{\theta }+\mathrm{RTln}\frac{1}{f_{Ce}\omega_{Ce}{\left(\omega_{O}\right)}^{\frac{3}{2}}}\;=\;-257.02\;\mathrm{KJ}/\mathrm{mol}<0$$

#### 3.1.3. Thermodynamic Properties of Cu-Ce-S System

The reaction between rare earth cerium and sulfur in a copper liquid at 1200 °C can be described as follows:

(15) $$\;[\mathrm{Ce}]{\mathrm{Cu}}_{\;}+[S]{\mathrm{Cu}}_{\;}=\mathrm{CeS}\;(s)\;∆G_{2}^{\theta }=\;-749,898\;+\;386.22T\;=\;180,995.94$$

Taking a 1% solution by mass as the standard state, according to Formulas (11)–(13),

(16) $$∆G_{2}\;=∆G_{2}^{\theta }\;+\mathrm{RTlnJ}=∆G_{2}^{\theta }\;+\mathrm{RTln}\frac{1}{f_{Ce}\omega_{Ce}f_{s}\omega_{s}}\;=\;-29.016\;\mathrm{KJ}/\mathrm{mol}\;<\;0$$

#### 3.1.4. Thermodynamic Properties of Cu-Ce-O-S System

The reaction of rare earth cerium with oxygen and sulfur in a copper liquid at 1200 °C is depicted as follows:

(17) $$[\mathrm{Ce}]{\mathrm{Cu}}_{\;}+[O{{]}_{\mathrm{Cu}}}_{\;}+\frac{1}{2}[S]{\mathrm{Cu}}_{\;}=\frac{1}{2}{\mathrm{Ce}}_{2}O_{2}S\;(s)\;∆G_{3}^{\theta }\;\;=\;1,027,589\;+\;410.84T\;=\;-422,421.68$$

Taking a 1% solution by mass as the standard state, according to Formulas (11)–(13),

(18) $$∆G_{3}=∆G_{3}^{\theta }\;+\mathrm{RTlnJ}=∆G_{3}^{\theta }+\mathrm{RTln}\frac{1}{f_{Ce}\omega_{Ce}{{\left(f_{s}\omega_{s}\right)}^{\frac{1}{2}}\omega }_{O}}\;=\;-218.517\;\mathrm{KJ}/\mathrm{mol}\;<\;0$$

The calculation results reveal that the values of $∆G_{1}$, $∆G_{2}$, and $∆G_{3}$ are negative, with significant magnitudes. This indicates a pronounced thermodynamic inclination for rare earth Ce to facilitate deoxidation and desulfurization reactions in copper, effectively removing trace amounts of O and S from the system.

### 3.2. Existing Forms of Rare Earth Ce in Copper

#### 3.2.1. Solid Solution of Ce in Copper

**Figure 1:**
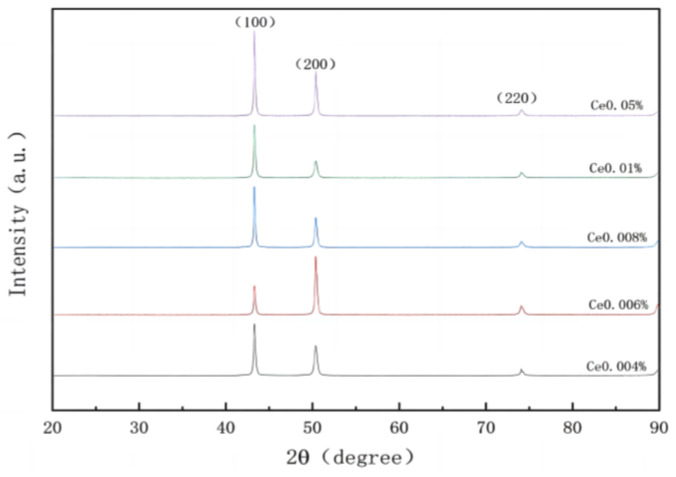
XRD diffraction stacking diagram of copper samples with different Ce contents.

Combined with the author’s previous research data, X-ray diffraction (XRD) analysis was conducted on samples containing varying amounts of Ce to investigate the correlation between diffraction peaks and angles for different elements and Ce addition. The obtained XRD diffraction pattern is presented in Figure 1. Due to the low concentration of the Ce element used in this experiment, only the diffraction peaks corresponding to Cu crystal planes (111), (200), and (220) are observed, while no discernible diffraction peaks related to the rare earth Ce element or its compounds are detected.

According to the relationship between the diffraction angle and content, a line diagram illustrating the correlation between the diffraction angle and content for different crystal planes is constructed.

**Figure 2:**
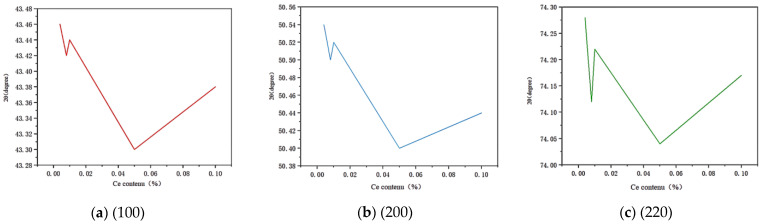
Variation curves of diffraction angle of different crystal planes.

In Figure 2, (a) represents the diffraction angle image of crystal plane (100), (b) represents the diffraction angle image of crystal plane (200), and (c) represents the diffraction angle image of crystal plane (220). According to Bragg’s law: 2dsinθ = nλ, the diffraction angle is inversely proportional to the spacing between crystal planes, while the lattice constant is positively correlated with this spacing. Therefore, a larger diffraction angle indicates a smaller lattice constant. Conversely, a larger lattice constant leads to increased solid solubility and a leftward shift of the diffraction peak. From Figure 2, it can be observed that within the Ce concentration range from 0.006% to 0.009%, there is a decrease in diffraction angles with increasing Ce content, accompanied by a gradual increase in the lattice constant, indicating a progressive dissolution of Ce into copper resulting in the formation of a solid solution between them. Within the range of 0.009% to 0.01% content, each grain boundary exhibits an upward trend in its respective diffraction angle, suggesting that Ce has reached its maximum solid solubility and started forming a supersaturated solid solution with copper leading to the precipitation of Ce elements as well. These findings demonstrate that Cu has an approximate maximum solid solubility for Ce within the range of 0.009~0.01%.

#### 3.2.2. Existing Forms of Ce in Pure Copper

**Figure 3:**
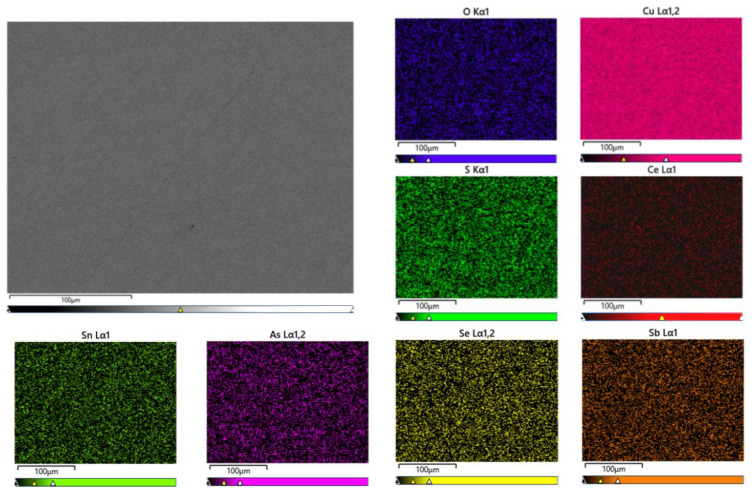
Mapping diagram of 2# Ce-containing copper sample matrix.

**Figure 4:**
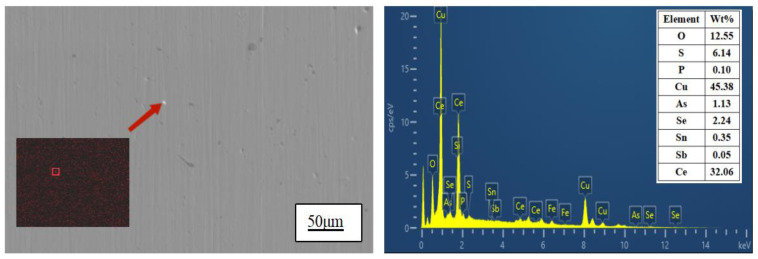
EDS analysis of typical compounds in matrix of 2# Ce-containing copper sample.

The surface of the sample was analyzed using a scanning electron microscope to further investigate the distribution and existing form of Ce in the matrix. As depicted in Figure 3, Ce was uniformly distributed within the Cu matrix in the sample with a Ce content of 0.0097%. However, aggregation was observed in the enlarged region indicated by the arrow, as shown in Figure 4.

EDS energy spectrum analysis was conducted in the region indicated by the arrow in Figure 4, revealing relatively high contents of Ce, Cu, O, and S elements. Thermodynamic calculations and analyses suggest that substances in this area may consist of Ce_2_O_3_, CeS, or Ce_2_O_2_S compounds; excessive amounts of Ce can also form intermetallic compounds with Cu. Zhang Shihong et al. [22] employed the Miedema thermodynamic model to compute that upon the addition of rare earth element Ce to purple bronze, Ce exhibits a preference for reacting with O and S elements, resulting in the formation of high-melting-point and low-density rare earth compounds. Simultaneously, rare earth Ce can also react with copper to generate corresponding copper/rare earth compounds, which disperse as second-phase particles within the copper matrix. The experimental results are consistent with these conclusions. During the solidification process, formed compounds serve as nucleation centers which facilitate microstructure refinement while pinning dislocations and improving the properties of the copper alloy.

#### 3.2.3. Effect of Rare Earth Ce on As-Cast Microstructure of Pure Copper

**Figure 5:**
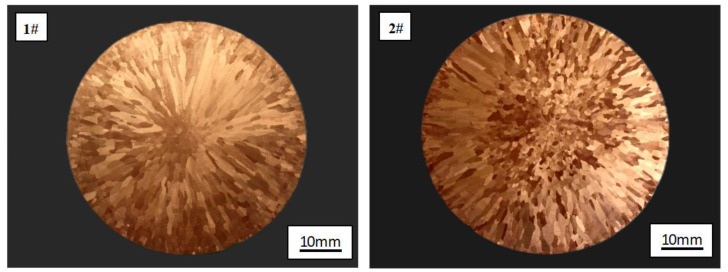
Transverse low magnification microstructure of 1# and 2# as-cast copper ingots.

The transverse low power structure of 1# and 2# as-cast copper ingots is depicted in Figure 5. It can be observed that the addition of rare earth significantly alters the microstructure of the as-cast copper ingot. In Figure 5, the majority of crystals from the edge to the center of the 1# copper ingot are columnar with coarse grains and uneven distribution, while a small number of equiaxed grains are present at the center. Upon adding rare earth, more equiaxed grains emerge at the center with enlarged size, and compared to those without rare earth addition, there is evident refinement in columnar grains at the edge.

The addition of rare earth effectively refines the as-cast grain of copper through a dual mechanism. Firstly, the inclusion of rare earth Ce reduces the melting temperature of pure copper, thereby increasing the undercooling degree of the composition. This increased undercooling promotes nucleation and improves the nucleation rate, subsequently inhibiting grain growth. Moreover, heightened undercooling in pure copper intensifies cellular dendrite growth and enhances dendritic development, ultimately resulting in reduced dendrite spacing and refined columnar crystals. Additionally, composition undercooling provides sufficient nucleation conditions for new equiaxed grains to form based on effective nucleation points created by rare earth atoms within this region. These newly formed equiaxed grains continue to grow while solute redistribution during grain growth generates another composition undercooling zone at the solid/liquid interface front surrounding grain growth. This facilitates continuous nucleation and growth at these sites, thus promoting the expansion of the equiaxed crystal region [21]. Secondly, upon adding rare earth to copper, preferential reactions between rare earth elements and other constituents lead to the formation of high-melting-point compounds which are finely dispersed throughout the molten copper matrix. During solidification, these fine high-melting-point compounds act as heterogeneous crystal nuclei that increase the crystal nucleus density while mechanically impeding grain growth processes. Consequently, this shortens solidification time and contracts the columnar crystal region [11].

#### 3.2.4. The Influence of Rare Earth Ce on Impurities in Pure Copper

**Figure 6:**
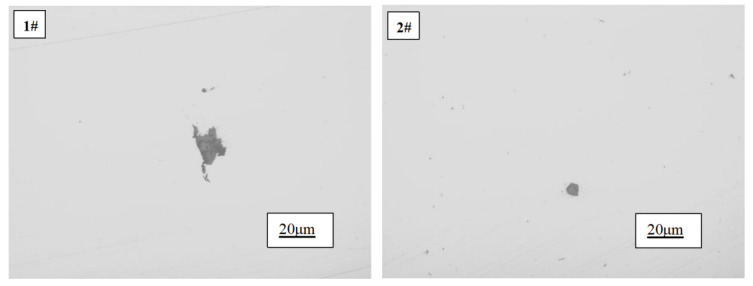
Morphology of inclusions in 1# and 2# as-cast copper ingots.

The overall morphology of inclusions in pure copper before and after the addition of rare earth is depicted in Figure 6. As illustrated by 1#, the inclusions present in pure copper ingots devoid of rare earth exhibit a substantial size and tend to aggregate, displaying an extremely irregular and angular morphology that significantly compromises the properties of copper. Conversely, as demonstrated by 2#, it is evident that the inclusion size becomes noticeably finer upon introducing a rare earth content of 97 ppm into the copper ingot. Moreover, their shape transforms from irregular to approximately round, while smaller-sized inclusions are uniformly dispersed throughout, indicating that rare earth has effectively enhanced the morphology of copper’s inclusions.

This result may be attributed to the following factors: Firstly, upon the addition of rare earths, they react with detrimental elements such as oxygen and sulfur in the copper liquid, forming high-melting-point inclusions. These inclusions preferentially precipitate, and some are entrained into the copper slag, leading to a reduction in impurities and the purification of the copper melt. Additionally, during this process, a small amount of high-melting-point oxides, sulfides, and other inclusions also precipitate from the copper liquid to form solid cores. Due to their strong reactivity, certain rare earths gradually adsorb onto these solid core surfaces. Simultaneously, owing to chemical potential gradients’ existence, there is a concentration disparity between nucleation centers and surrounding rare earth atoms that drives more rare earth atoms towards nucleation cores. Through continuous attraction, convergence, and fusion processes, rare earth oxide/sulfide compounds eventually form whose size depends on the aggregation level of rare earth elements. Since there is relatively less presence of rare earth elements compared to oxygen or sulfides at this stage, the size of inclusions remains relatively small. Secondly, the addition of rare earths alters inclusion types resulting in improved morphology.

### 3.3. Effect of Rare Earth Ce on Mechanical Properties of Copper

#### 3.3.1. Effect of Rare Earth Ce on Mechanical Properties of Copper

The mechanical properties of copper, both before and after the addition of rare earth Ce, are presented in Table 3. It is evident that the incorporation of Ce leads to enhancements in both the tensile strength and elongation of copper. Specifically, the average tensile strength and elongation values for copper with Ce addition are measured at 154 MPa and 33%, respectively, representing an increase of 8.45% compared to pure copper’s tensile strength and a remarkable improvement by 12.1% over its elongation.

**Figure 7:**
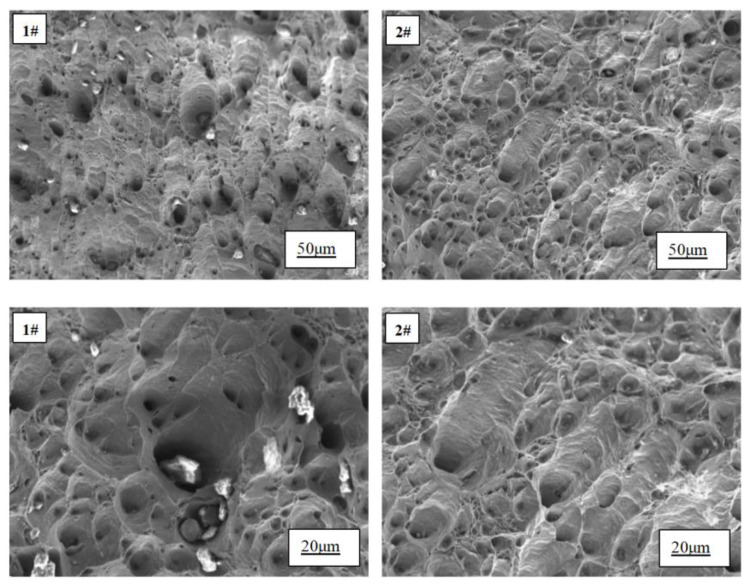
Tensile fracture morphology of 1# and 2# copper samples.

The tensile fracture morphology before and after the addition of Ce is presented in Figure 7. It can be observed that both samples exhibit ductile fracture characteristics. However, upon the addition of rare earth, an increase in the number of dimples on the fracture surface is evident, accompanied by a reduction in their size and a more uniform distribution. In contrast, without the addition of rare earth, numerous irregularly shaped and large-sized inclusions surround the dimples on the fracture surface. Remarkably, with the incorporation of rare earth, there is a noticeable decrease in inclusion content. Upon magnification, smaller round- or oval-shaped inclusions within dimples are uniformly distributed.

When the specimen undergoes tensile deformation, the grain boundary serves as a region of stress concentration. With continuous tensile loading, micro-holes tend to form at the grain boundary. These micro-holes act as nucleation sites for dimples and gradually expand, eventually leading to fracture in the copper grain structure. The presence of larger-size inclusions increases the propensity for fracture, which contributes to premature failure in samples without rare earth addition. Upon introducing rare earth Ce, two significant improvements are observed: Firstly, the refinement of grains occurs due to its addition; according to the Hall–Petch relationship [23], refining grains can substantially enhance a material’s tensile strength by negatively correlating it with grain size. Secondly, rare earth Ce enhances inclusion morphology by transforming large-size irregular inclusions into fine and spheroidized ones that are uniformly dispersed throughout the material matrix. These finely distributed inclusions effectively impede dislocation movement and improve deformation resistance, thereby enhancing material strength.

#### 3.3.2. Effect of Ce on Hardness

**Figure 8:**
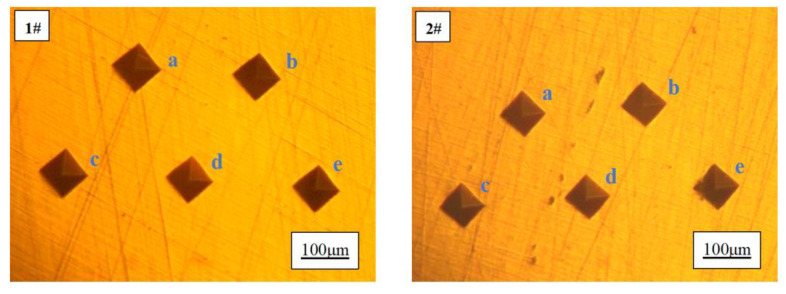
Microhardness diagram of 1# and 2# copper samples.

The microhardness tests were conducted on Sample 1 and Sample 2 at five different testing locations, namely positions a, b, c, d, and e as depicted in Figure 8. Based on the test results presented in Table 4, it is evident that: After the addition of rare earth Ce, the hardness of pure copper samples increased from 73.5 HV to 81.2 HV, representing a significant enhancement of 10.5%. When subjected to pressure load, pure copper undergoes deformation accompanied by the extensive movement and slip of dislocations within its structure. The presence of a finer grain size results in higher grain boundary density and a greater accumulation of additional dislocations at these boundaries, thereby increasing resistance against external forces and elevating material hardness. Additionally, the finely dispersed rare earth inclusions impede dislocation motion and further enhance material deformation resistance [24,25,26]. This phenomenon elucidates the rationale behind the observed increase in hardness upon incorporating rare earth Ce into pure copper.

## 4. Conclusions

(1) By thermodynamically analyzing the mutual conversion of reaction products in this experiment, it has been determined that the final inclusion products of rare earth Ce in copper melt at 1200 °C consist of Ce_2_O_3_ (s), Ce_2_O_2_S (s), and CeS (s).

(2) The thermodynamic calculations reveal that the Gibbs free energy of the reaction between rare earth element Ce and oxygen (O) as well as sulfur (S) in liquid copper at 1200 °C is negative, indicating a strong thermodynamic driving force for the deoxidation and desulfurization of Ce in copper. Consequently, the resulting products include Ce_2_O_3_, CeS, and CeSO.

(3) The XRD analysis results demonstrate an increase in the diffraction angle of each grain boundary when the concentration of Ce in Cu ranges from 0.009% to 0.01%. In accordance with Bragg’s law, the maximum solid solubility of Ce in Cu falls within the range of 0.009% to 0.01%.

(4) After the addition of Ce, equiaxed grains emerge at the core of the as-cast copper ingot, while the columnar grains at the periphery undergo evident refinement. This observation demonstrates that rare earth Ce facilitates the transition from columnar to equiaxed grain morphology and refines the microstructure of as-cast copper.

(5) After the incorporation of rare earth elements, the tensile strength, elongation, and microhardness of pure copper exhibited a significant enhancement by 8.45%, 12.1%, and 10.5%, respectively, resulting in an increase from 73.5 HV to 81.2 HV. It is worth noting that the addition of rare earth element Ce demonstrated a pronounced improvement in the mechanical properties of cast pure copper.

## Figures and Tables

**Table 1:** The reaction and activity products of rare earth Ce with O and S in a copper solution.

Reaction and Products	$∆G^{\theta }$ (J/mol)	$\mathrm{Activity}\;\mathrm{Product}\;\prod a_{i}$
[Ce] + 2[O] = CeO_2_ (S)	−1,228,708 + 474.78T	$\prod a_{1}\;$= 5.34 × 10^−19^
[Ce] +$\frac{3}{2}$[O] = $\frac{1}{2}$Ce_2_O_3_ (S)	−1,074,469 + 415.28T	$\prod a_{2}\;$= 3.88 × 10^−17^
[Ce] + [O] +$\frac{1}{2}$[S] $=\frac{1}{2}$Ce_2_O_2_S (S)	−1,027,589 + 410.84T	$\prod a_{3}$ = 5.867 × 10^−17^
[Ce] + [S] = CeS (S)	−749,898 + 386.2T	$\prod a_{4}\;$= 3.89 × 10^−7^
[Ce] +$\frac{3}{2}$[S] = $\frac{1}{2}$Ce_2_S_3_ (S)	−871,909 + 418.8T	$\prod a_{5}\;$= 3.01 × 10^−7^
[Ce] +$\frac{4}{3}$[S] = $\frac{1}{3}$Ce_3_O_4_ (S)	−830,793 + 411.55T	$\prod a_{6}$ = 1.08 × 10^−5^

**Table 2:** Interaction coefficients between main elements in copper.

$e_{i}^{j}$	Ce	S	O
Ce	0.108	−37.92	−0.725
S	−5.36	−0.143	0.33
O	−0.083	−0.164	−0.169

**Table 3:** Mechanical properties of 1# and 2# copper samples.

Sample Number	Ce Content, wt.%	Rm, Mpa	Average, Mpa	A, %	Average, %
1#	0	139	142	34	33
		144		33	
		143		32	
2#	0.0097	154	154	39	37
		155		36	
		153		36	

**Table 4:** Microhardness values of 1# and 2# copper samples.

Sample Number	Ce Content, wt.%	Hardness, HV	Average, HV
1#	0	a: 75.4; b: 75.9; c: 71.5; d: 72.1; e: 72.8	73.5
2#	0.0097	a: 80.7; b: 81.8; c: 80.1; d: 81.8; e: 81.5	81.2

## Data Availability

Data are contained within the article.
